# Supplementary material for: Hot spot prediction in protein-protein interactions by an ensemble system
Source: BMC Syst Biol. 2018 Dec 31;12(Suppl 9):132. doi: 10.1186/s12918-018-0665-8 (PMC6311905; doi:10.1186/s12918-018-0665-8)
Supplement: Supplementary file 2 — All relevant MATLAB code. A simple Matlab implement of our predictor. (ZIP 19,742 kb) [file 12918_2018_665_MOESM2_ESM.zip › Additional file 2/Read ASA/ASA website/dbMPIKT/4BFIA.html]

 NetSurfP - Protein Surface Accessibility and Secondary Structure Predictions

  

|  |  |  |
| --- | --- | --- |
|  |  | NetSurfP - Protein Surface Accessibility and Secondary Structure Predictions  Technical University of Denmark |

  


---

```
# For publication of results, please cite:
# A generic method for assignment of reliability scores applied to solvent accessibility predictions.
# Bent Petersen, Thomas Nordahl Petersen, Pernille Andersen, Morten Nielsen and Claus Lundegaard
# BMC Structural Biology 2009, 9:51 doi:10.1186/1472-6807-9-51
#
# Column 1: Class assignment - B for buried or E for Exposed - Threshold: 25% exposure, but not based on RSA
# Column 2: Amino acid
# Column 3: Sequence name
# Column 4: Amino acid number
# Column 5: Relative Surface Accessibility - RSA
# Column 6: Absolute Surface Accessibility
# Column 7: Z-fit score for RSA prediction
# Column 8: Probability for Alpha-Helix
# Column 9: Probability for Beta-strand
# Column 10: Probability for Coil
E T  4BFIA                  1    0.705  97.742  -0.402   0.003   0.003   0.994
E D  4BFIA                  2    0.511  73.635  -1.941   0.005   0.045   0.951
E K  4BFIA                  3    0.582 119.800  -0.569   0.018   0.088   0.893
E N  4BFIA                  4    0.549  80.432   0.576   0.005   0.262   0.733
E Q  4BFIA                  5    0.289  51.669   0.727   0.003   0.718   0.279
E T  4BFIA                  6    0.328  45.494   0.713   0.001   0.900   0.099
B T  4BFIA                  7    0.178  24.716   0.059   0.001   0.959   0.040
E Q  4BFIA                  8    0.320  57.134   0.218   0.001   0.900   0.099
B N  4BFIA                  9    0.198  28.987  -0.926   0.002   0.816   0.182
E N  4BFIA                 10    0.462  67.578  -0.076   0.004   0.514   0.481
E S  4BFIA                 11    0.438  51.345  -1.197   0.004   0.138   0.858
E S  4BFIA                 12    0.618  72.406  -1.283   0.004   0.085   0.910
E S  4BFIA                 13    0.525  61.577  -1.414   0.005   0.262   0.733
E P  4BFIA                 14    0.429  60.875  -0.955   0.004   0.514   0.481
E L  4BFIA                 15    0.434  79.429  -0.894   0.002   0.816   0.182
E T  4BFIA                 16    0.388  53.802  -0.595   0.002   0.816   0.182
E Q  4BFIA                 17    0.565 100.945   0.134   0.002   0.816   0.182
E V  4BFIA                 18    0.321  49.276   0.538   0.004   0.616   0.381
E N  4BFIA                 19    0.583  85.380  -0.123   0.005   0.336   0.660
E T  4BFIA                 20    0.487  67.575   0.228   0.004   0.420   0.576
E T  4BFIA                 21    0.560  77.727   0.719   0.003   0.718   0.279
B V  4BFIA                 22    0.079  12.065   0.556   0.001   0.900   0.099
E S  4BFIA                 23    0.458  53.678   1.262   0.011   0.918   0.071
B V  4BFIA                 24    0.066  10.129  -0.239   0.001   0.900   0.099
E Q  4BFIA                 25    0.416  74.298   0.943   0.002   0.816   0.182
E I  4BFIA                 26    0.329  60.884   0.618   0.005   0.336   0.660
E G  4BFIA                 27    0.569  44.772  -0.420   0.005   0.045   0.951
E T  4BFIA                 28    0.608  84.371   0.307   0.005   0.045   0.951
E K  4BFIA                 29    0.619 127.328   0.899   0.005   0.336   0.660
B A  4BFIA                 30    0.066   7.284  -0.278   0.002   0.816   0.182
E L  4BFIA                 31    0.466  85.251   1.158   0.001   0.900   0.099
B L  4BFIA                 32    0.063  11.499   0.276   0.001   0.900   0.099
E C  4BFIA                 33    0.446  62.590   0.636   0.004   0.616   0.381
B C  4BFIA                 34    0.058   8.115  -0.357   0.004   0.420   0.576
E F  4BFIA                 35    0.469  94.108  -0.024   0.004   0.514   0.481
B S  4BFIA                 36    0.252  29.570  -1.625   0.004   0.616   0.381
E I  4BFIA                 37    0.443  81.881  -0.028   0.004   0.514   0.481
E P  4BFIA                 38    0.462  65.515  -1.516   0.021   0.279   0.699
E L  4BFIA                 39    0.436  79.795  -1.212   0.018   0.088   0.893
E T  4BFIA                 40    0.492  68.268  -1.367   0.004   0.085   0.910
E K  4BFIA                 41    0.614 126.238  -0.312   0.004   0.197   0.799
E A  4BFIA                 42    0.327  35.991  -1.203   0.004   0.514   0.481
E V  4BFIA                 43    0.294  45.157  -0.331   0.003   0.718   0.279
E L  4BFIA                 44    0.358  65.605   0.612   0.001   0.900   0.099
B I  4BFIA                 45    0.037   6.845   0.310   0.001   0.959   0.040
B T  4BFIA                 46    0.227  31.485   0.906   0.001   0.959   0.040
B W  4BFIA                 47    0.070  16.883   0.534   0.001   0.959   0.040
E I  4BFIA                 48    0.285  52.743   1.095   0.001   0.959   0.040
B I  4BFIA                 49    0.108  19.980   0.386   0.001   0.959   0.040
E K  4BFIA                 50    0.422  86.723   0.351   0.001   0.900   0.099
B L  4BFIA                 51    0.288  52.824  -0.815   0.004   0.514   0.481
E R  4BFIA                 52    0.600 137.308  -0.668   0.005   0.045   0.951
E G  4BFIA                 53    0.758  59.686  -1.938   0.003   0.003   0.994
E L  4BFIA                 54    0.547 100.082  -0.746   0.005   0.015   0.979
E P  4BFIA                 55    0.334  47.395  -0.609   0.005   0.045   0.951
E S  4BFIA                 56    0.505  59.233   0.463   0.005   0.262   0.733
B C  4BFIA                 57    0.157  22.071  -0.141   0.003   0.718   0.279
E T  4BFIA                 58    0.271  37.560   0.711   0.001   0.900   0.099
B I  4BFIA                 59    0.072  13.302   0.848   0.001   0.959   0.040
B A  4BFIA                 60    0.225  24.751   1.051   0.001   0.959   0.040
B Y  4BFIA                 61    0.061  13.078   0.476   0.001   0.959   0.040
E K  4BFIA                 62    0.272  56.033   0.791   0.001   0.900   0.099
E V  4BFIA                 63    0.379  58.283   0.373   0.004   0.420   0.576
E D  4BFIA                 64    0.687  98.997  -0.927   0.018   0.019   0.964
E T  4BFIA                 65    0.595  82.540  -1.535   0.018   0.047   0.935
E K  4BFIA                 66    0.559 114.904  -0.331   0.004   0.514   0.481
E T  4BFIA                 67    0.471  65.369  -1.120   0.023   0.655   0.322
E N  4BFIA                 68    0.390  57.096  -0.564   0.023   0.655   0.322
E E  4BFIA                 69    0.491  85.725  -1.004   0.021   0.451   0.528
E T  4BFIA                 70    0.401  55.646  -1.551   0.020   0.205   0.775
E S  4BFIA                 71    0.499  58.483  -1.590   0.018   0.088   0.893
E C  4BFIA                 72    0.538  75.479  -1.350   0.018   0.088   0.893
E L  4BFIA                 73    0.416  76.261  -1.348   0.019   0.141   0.840
E G  4BFIA                 74    0.542  42.624  -1.452   0.004   0.138   0.858
E R  4BFIA                 75    0.437 100.073  -0.621   0.005   0.336   0.660
B N  4BFIA                 76    0.182  26.616  -0.579   0.002   0.816   0.182
B I  4BFIA                 77    0.087  16.021  -0.270   0.001   0.959   0.040
B T  4BFIA                 78    0.217  30.126  -0.236   0.001   0.959   0.040
B W  4BFIA                 79    0.134  32.251   0.057   0.001   0.959   0.040
B A  4BFIA                 80    0.224  24.729  -0.392   0.001   0.900   0.099
E S  4BFIA                 81    0.372  43.598  -1.316   0.003   0.718   0.279
E T  4BFIA                 82    0.406  56.284  -0.848   0.004   0.420   0.576
E P  4BFIA                 83    0.594  84.289  -1.503   0.004   0.085   0.910
E D  4BFIA                 84    0.548  79.024  -1.802   0.005   0.045   0.951
E H  4BFIA                 85    0.607 110.413  -1.184   0.004   0.085   0.910
E S  4BFIA                 86    0.543  63.675  -0.247   0.005   0.262   0.733
B P  4BFIA                 87    0.090  12.743  -0.207   0.004   0.616   0.381
B E  4BFIA                 88    0.220  38.451   0.616   0.001   0.900   0.099
B L  4BFIA                 89    0.026   4.724   0.783   0.001   0.959   0.040
E Q  4BFIA                 90    0.256  45.650   1.683   0.001   0.959   0.040
B I  4BFIA                 91    0.034   6.309   0.245   0.001   0.900   0.099
E S  4BFIA                 92    0.453  53.092   1.377   0.003   0.718   0.279
E A  4BFIA                 93    0.579  63.828   0.815   0.004   0.514   0.481
B V  4BFIA                 94    0.106  16.261   0.720   0.004   0.420   0.576
E T  4BFIA                 95    0.483  67.034   0.934   0.004   0.420   0.576
E L  4BFIA                 96    0.526  96.366   0.483   0.005   0.336   0.660
E Q  4BFIA                 97    0.575 102.784   0.020   0.004   0.138   0.858
B H  4BFIA                 98    0.289  52.496  -0.257   0.004   0.085   0.910
E E  4BFIA                 99    0.626 109.345   0.379   0.004   0.138   0.858
B G  4BFIA                100    0.164  12.867  -1.436   0.003   0.718   0.279
E T  4BFIA                101    0.418  58.004   1.968   0.001   0.959   0.040
B Y  4BFIA                102    0.045   9.510   0.794   0.001   0.959   0.040
E T  4BFIA                103    0.264  36.575   2.299   0.001   0.959   0.040
B C  4BFIA                104    0.014   2.008   1.608   0.001   0.959   0.040
E E  4BFIA                105    0.252  44.042   2.127   0.001   0.959   0.040
B T  4BFIA                106    0.042   5.853   0.155   0.001   0.959   0.040
E V  4BFIA                107    0.421  64.738   0.908   0.001   0.959   0.040
B T  4BFIA                108    0.118  16.367   0.058   0.003   0.718   0.279
E P  4BFIA                109    0.662  93.980   0.043   0.004   0.085   0.910
E E  4BFIA                110    0.601 105.082  -1.036   0.004   0.085   0.910
E G  4BFIA                111    0.427  33.597  -1.220   0.004   0.420   0.576
E N  4BFIA                112    0.505  73.873  -0.627   0.002   0.816   0.182
E F  4BFIA                113    0.340  68.198  -0.413   0.001   0.900   0.099
E E  4BFIA                114    0.478  83.489  -0.052   0.001   0.900   0.099
B K  4BFIA                115    0.189  38.857   0.019   0.001   0.959   0.040
E N  4BFIA                116    0.374  54.768   0.810   0.001   0.959   0.040
B Y  4BFIA                117    0.063  13.399   0.374   0.001   0.959   0.040
E D  4BFIA                118    0.315  45.392   1.598   0.001   0.959   0.040
B L  4BFIA                119    0.024   4.376   0.914   0.001   0.959   0.040
E Q  4BFIA                120    0.298  53.187   1.831   0.001   0.959   0.040
B V  4BFIA                121    0.038   5.887   0.234   0.001   0.959   0.040
E L  4BFIA                122    0.325  59.581   0.914   0.001   0.900   0.099
B V  4BFIA                123    0.182  27.912   0.303   0.003   0.718   0.279
E P  4BFIA                124    0.416  59.016   0.773   0.004   0.138   0.858
B P  4BFIA                125    0.139  19.710  -0.451   0.004   0.197   0.799
E E  4BFIA                126    0.568  99.299   0.531   0.004   0.616   0.381
B V  4BFIA                127    0.219  33.599  -0.144   0.001   0.900   0.099
E T  4BFIA                128    0.411  57.006   0.430   0.001   0.900   0.099
B Y  4BFIA                129    0.142  30.388  -0.141   0.002   0.816   0.182
E F  4BFIA                130    0.465  93.305   0.967   0.004   0.514   0.481
E P  4BFIA                131    0.560  79.436  -0.253   0.004   0.138   0.858
E E  4BFIA                132    0.727 126.972  -0.596   0.005   0.015   0.979
E K  4BFIA                133    0.576 118.483   0.237   0.005   0.045   0.951
E N  4BFIA                134    0.589  86.200   0.944   0.004   0.420   0.576
B R  4BFIA                135    0.067  15.274   0.309   0.001   0.900   0.099
E S  4BFIA                136    0.393  46.106   1.623   0.001   0.959   0.040
B A  4BFIA                137    0.042   4.672   0.679   0.001   0.959   0.040
E V  4BFIA                138    0.333  51.213   1.463   0.001   0.959   0.040
B C  4BFIA                139    0.022   3.131   0.744   0.001   0.959   0.040
E E  4BFIA                140    0.407  71.120   1.356   0.001   0.959   0.040
B A  4BFIA                141    0.111  12.232  -0.765   0.002   0.816   0.182
E M  4BFIA                142    0.530 106.093  -0.099   0.004   0.420   0.576
E A  4BFIA                143    0.699  77.008  -1.194   0.005   0.045   0.951
E G  4BFIA                144    0.416  32.700  -1.083   0.005   0.045   0.951
E K  4BFIA                145    0.502 103.282   0.140   0.004   0.138   0.858
B P  4BFIA                146    0.159  22.590  -0.548   0.004   0.138   0.858
E A  4BFIA                147    0.467  51.507   1.190   0.005   0.336   0.660
B A  4BFIA                148    0.094  10.370  -0.432   0.004   0.616   0.381
E Q  4BFIA                149    0.421  75.191   1.195   0.001   0.900   0.099
B I  4BFIA                150    0.044   8.103   0.365   0.001   0.959   0.040
E S  4BFIA                151    0.339  39.684   1.788   0.001   0.959   0.040
B W  4BFIA                152    0.035   8.345   0.046   0.001   0.959   0.040
E S  4BFIA                153    0.310  36.320   1.327   0.001   0.959   0.040
E P  4BFIA                154    0.312  44.244   1.236   0.003   0.718   0.279
E D  4BFIA                155    0.701 101.057  -0.095   0.018   0.019   0.964
E G  4BFIA                156    0.711  55.964  -1.936   0.016   0.005   0.979
E D  4BFIA                157    0.696 100.351   0.530   0.004   0.514   0.481
E C  4BFIA                158    0.523  73.415   0.242   0.003   0.718   0.279
B V  4BFIA                159    0.190  29.249   0.175   0.021   0.451   0.528
E T  4BFIA                160    0.491  68.088   0.098   0.021   0.279   0.699
E T  4BFIA                161    0.546  75.661  -0.460   0.020   0.205   0.775
E S  4BFIA                162    0.558  65.409  -1.031   0.021   0.279   0.699
E E  4BFIA                163    0.583 101.815  -0.675   0.023   0.655   0.322
E S  4BFIA                164    0.585  68.515  -0.421   0.023   0.655   0.322
E H  4BFIA                165    0.337  61.282  -0.605   0.005   0.336   0.660
E S  4BFIA                166    0.706  82.743  -1.663   0.004   0.138   0.858
E N  4BFIA                167    0.646  94.574  -1.375   0.005   0.045   0.951
E G  4BFIA                168    0.307  24.122  -0.965   0.005   0.262   0.733
E T  4BFIA                169    0.345  47.893   0.279   0.002   0.816   0.182
B V  4BFIA                170    0.130  19.935   0.681   0.001   0.959   0.040
B T  4BFIA                171    0.167  23.163  -0.108   0.001   0.959   0.040
B V  4BFIA                172    0.079  12.158   0.145   0.001   0.959   0.040
B R  4BFIA                173    0.248  56.678   0.031   0.001   0.959   0.040
B S  4BFIA                174    0.157  18.436  -1.045   0.001   0.959   0.040
E T  4BFIA                175    0.271  37.629   0.174   0.001   0.959   0.040
B C  4BFIA                176    0.185  25.918   0.390   0.001   0.959   0.040
E H  4BFIA                177    0.437  79.563   0.645   0.001   0.900   0.099
B W  4BFIA                178    0.087  20.948   0.513   0.003   0.718   0.279
E E  4BFIA                179    0.492  85.987   0.926   0.004   0.616   0.381
E Q  4BFIA                180    0.538  96.140  -0.016   0.005   0.262   0.733
E N  4BFIA                181    0.553  80.959  -0.528   0.005   0.045   0.951
E N  4BFIA                182    0.594  86.918  -0.660   0.005   0.015   0.979
E V  4BFIA                183    0.569  87.471  -0.097   0.004   0.085   0.910
B S  4BFIA                184    0.237  27.730  -0.354   0.004   0.514   0.481
E D  4BFIA                185    0.437  62.972   1.823   0.001   0.900   0.099
B V  4BFIA                186    0.048   7.408   0.895   0.001   0.959   0.040
B S  4BFIA                187    0.252  29.546   1.612   0.001   0.959   0.040
B C  4BFIA                188    0.016   2.190   1.377   0.001   0.959   0.040
B I  4BFIA                189    0.227  41.958   1.878   0.001   0.959   0.040
B V  4BFIA                190    0.030   4.611  -0.062   0.001   0.959   0.040
E S  4BFIA                191    0.406  47.607   0.904   0.001   0.959   0.040
B H  4BFIA                192    0.108  19.718   0.316   0.004   0.616   0.381
E L  4BFIA                193    0.651 119.143   1.018   0.018   0.047   0.935
E T  4BFIA                194    0.442  61.333  -0.661   0.004   0.085   0.910
E G  4BFIA                195    0.484  38.091  -0.744   0.005   0.262   0.733
E N  4BFIA                196    0.591  86.464   0.046   0.004   0.420   0.576
E Q  4BFIA                197    0.498  88.925   0.654   0.002   0.816   0.182
E S  4BFIA                198    0.548  64.237   0.606   0.001   0.900   0.099
B L  4BFIA                199    0.191  34.990   0.708   0.001   0.959   0.040
E S  4BFIA                200    0.445  52.119   0.867   0.001   0.959   0.040
B I  4BFIA                201    0.047   8.787   0.542   0.001   0.959   0.040
E E  4BFIA                202    0.438  76.431   1.683   0.001   0.959   0.040
B L  4BFIA                203    0.053   9.649   0.417   0.001   0.900   0.099
E G  4BFIA                204    0.277  21.808   0.404   0.004   0.616   0.381
E R  4BFIA                205    0.452 103.462  -0.209   0.022   0.359   0.619
E G  4BFIA                206    0.499  39.295  -0.966   0.020   0.205   0.775
E G  4BFIA                207    0.460  36.218  -1.375   0.020   0.205   0.775
E S  4BFIA                208    0.540  63.347  -0.119   0.020   0.205   0.775
B T  4BFIA                209    0.298  41.277  -0.510   0.018   0.088   0.893
E R  4BFIA                210    0.401  91.760  -0.161   0.018   0.088   0.893
E H  4BFIA                211    0.420  76.398  -0.379   0.019   0.141   0.840
E H  4BFIA                212    0.523  95.170  -0.840   0.019   0.141   0.840
E H  4BFIA                213    0.438  79.690  -1.045   0.021   0.279   0.699
E H  4BFIA                214    0.378  68.704  -0.787   0.021   0.279   0.699
E H  4BFIA                215    0.511  92.969  -0.838   0.020   0.205   0.775
E H  4BFIA                216    0.724 131.641  -0.902   0.003   0.003   0.994

Explain the output.  Go back.
```

---
